# Supplementary material for: Construction of a Chikungunya Virus, Replicon, and Helper Plasmids for Transfection of Mammalian Cells
Source: Viruses. 2022 Dec 31;15(1):132. doi: 10.3390/v15010132 (PMC9864538; doi:10.3390/v15010132)

Supplementary information

Table S1. Primers used to clone the plasmids

Primers	Sequence 5´to 3´
SP6toBamHI-F	GCTCCTGGATCCAGGTGACAC
SP6toBamHI-R	GTGTCACCTGGATCCAGGAGC
DelNsP4-F	CATGGCCACCTTTGCAAG
DelNsP1-R	CTTCCTGTCCGACATCATC
DelCPE3-F	GCCATTATGGTGAGCGAG
DelCPE3-R	CTCCATTATGGCTGATTGG
DelFMtoE1-F	CTTGACAACTAGGTACGAAGGTA
DelFMtoE1-R	TCTGTGCCCCAGTTTGCTA
ICD-F	AAAAAAAAAAAAAAAAGGGTCGGCATGGCATC
ICD-R	GTCTCACGCAGCCATACGGTTCACTAAACG
Chik-F	CGTTTAGTGAACCGTATGGCTGCGTGAGAC
Chik-R	ATGCCATGCCGACCCTTTTTTTTTTTTTTTTTTTTTTTTT TTGAAATATT

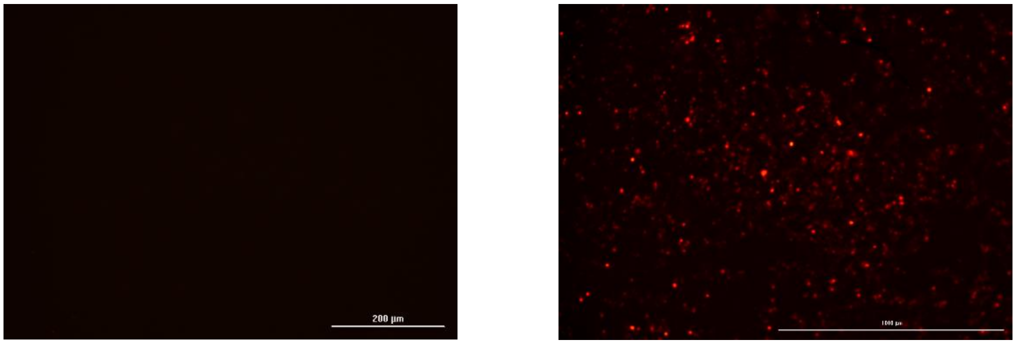

(**a**) (**b**)

Figure S1. Micrographs of HEK-293T cells transfected in fluorescent field (Texas Red filter) with 1 µg of the plasmid: (**a**) pVax-Rep, bar 200 µm; (**b**) pVax-Help, bar 1000 µm.

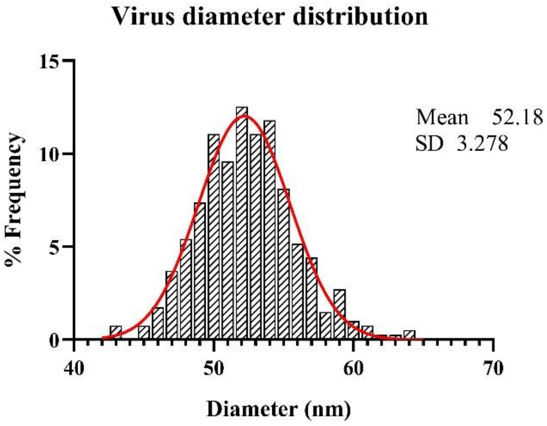

Figure S2. Histograms of CHIKV particles diameter distribution measure by transmission electron microscopy.

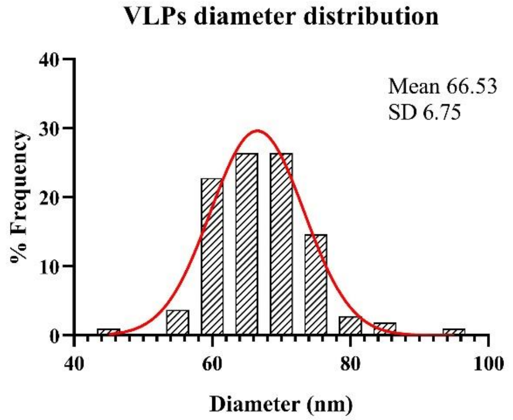

Figure S3. Histograms of VLPs diameter distribution measure by transmission electron microscopy.

pACNR-CHIKV plasmid sequence

ATGGCTGCGTGAGACACACGTAGCCTACCAGTTTCTTACTGCTCTACTCTGCAAAGCAAGAGATTAATAAC
CCATCATGGATTCTGTGTACGTGGACATAGACGCTGACAGCGCCTTTTTGAAGGCCCTGCAACGTGCGTAC
CCCATGTTTGAGGTGGAACCTAGGCAGGTCACATCGAATGACCATGCTAATGCTAGAGCGTTCTCGCATCT
AGCCATAAAACTAATAGAGCAGGAAATTGATCCCGACTCAACCATCCTGGATATAGGTAGTGCGCCAGCA
AGGAGGATGATGTCGGACAGGAAGTACCACTGCGTTTGCCCGATGCGCAGCGCAGAAGATCCCGAGAGA
CTCGCTAATTATGCGAGAAAGCTCGCATCTGCCGCAGGAAAAGTCCTGGACAGAAACATTTCTGGAAAGA
TCGGGGACTTACAAGCGGTGATGGCCGTGCCAGACACGGAGACGCCAACATTTTGCTTACACACAGATGT
CTCATGTAGACAGAGAGCAGACGTCGCGATATACCAAGACGTCTATGCTGTACACGCACCCACGTCGCTA
TACCACCAGGCGATTAAAGGAGTCCGAGTGGCGTACTGGGTAGGGTTCGACACAACCCCGTTCATGTACA
ACGCTATGGCGGGTGCCTACCCCTCATACTCGACAAATTGGGCGGATGAGCAGGTACTGAAGGCTAAGAA
CATAGGATTATGTTCAACAGACCTGACGGAAGGTAGACGAGGCAAATTGTCTATCATGAGAGGGAAAAAG
CTAAAACCGTGCGACCGTGTGCTGTTCTCAGTAGGGTCAACGCTTTACCCGGAAAGCCGCACGCTACTTAA
GAGCTGGCACCTACCATCGGTGTTCCATCTAAAGGGCAAGCTTAGCTTCACATGCCGCTGTGACACAGTGG
TTTCGTGTGAGGGCTACGTCGTTAAGAGAATAACGATGAGCCCAGGCCTTTATGGAAAAACCATAGGGTAT
GCGGTAACCCACCACGCAGACGGATTCTTGATGTGCAAGACTACCGACACGGTTGACGGCGAAAGAGTGT
CATTCTCGGTGTGCACGTACGTGCCGGCGACCATTTGTGATCAAATGACCGGCATCCTTGCTACAGAAGTC
ACGCCGGAGGATGCACAGAAGCTGTTGGTGGGGCTGAACCAGAGGATAGTGGTTAACGGCAGAACGCAA
CGGAACACGAACACCATGAAGAACTACCTACTTCCCGTGGTCGCCCAGGCCTTCAGTAAGTGGGCAAAGG
AGTGCCGGAAGGACATGGAAGATGAGAAGCTTCTGGGGGTCAGAGAAAGAACACTAACCTGCTGCTGTCT
ATGGGCATTTAAGAAGCAGAAAACACACACGGTCTACAAGAGGCCTGATACCCAGTCAATCCAGAAGGTT
CAGGCCGAATTTGACAGCTTTGTAGTACCGGGCCTGTGGTCGTCCGGGTTGTCAATCCCGTTGAGGACTAG
AATCAAGTGGTTGTTACGCAAGGTGCCGAAAACAGACCTGATCCCATACAGCGGGAATGCCCAAGAAGCC
CAGGATGCAGAAAAAGAAGCAGAGGAAGAACGAGAAGCAGAACTGACTCATGAGGCTCTACCACCCCTA
CAGGCAGCACAGGAAGATGTCCAGGTCGAAATCGACGTGGAACAGCTTGAGGATAGAGCTGGTGCTGGA
ATAATAGAGACTCCGAGAGGCGCTATCAAAGTTACTGCCCAACTAACAGACCACGTCGTGGGGGAGTACC

TGGTACTTTCCCCGCAGACCGTACTACGCAGCCAGAAGCTCAGCCTGATCCACGCTTTAGCGGAGCAAGT
GAAGACGTGTACGCACAGCGGACGAGCAGGGAGGTATGCGGTCGAAGCGTACGATGGCCGAGTCCTAGT
GCCCTCAGGCTATGCAATTTCGCCTGAAGACTTCCAGAGTCTAAGCGAAAGCGCAACGATGGTGTACAAC
GAAAGAGAGTTCGTAAACAGAAAGTTACACCACATTGCGATGCACGGACCAGCCCTGAACACTGACGAA
GAGTCGTATGAGCTGGTGAGGGCAGAGAGGACAGAACACGAGTACGTCTACGACGTGGACCAGAGAAGA
TGCTGTAAGAAGGAAGAAGCTGCAGGACTGGTACTGGTGGGCGACTTGACTAATCCGCCCTACCACGAAT
TCGCATACGAAGGGCTAAAAATTCGCCCCGCCTGCCCATACAAAATTGCAGTCATAGGAGTCTTCGGGGTA
CCAGGATCTGGCAAGTCAGCCATTATCAAGAACCTAGTTACCAGGCAAGACCTGGTGACTAGCGGAAAGA
AAGAAAACTGCCAAGAAATCAGCACCGACGTGATGAGACAGAGAGGTCTAGAGATATCTGCACGTACGG
TAGATTCGCTGCTCTTGAATGGATGCAACAGACCAGTCGACGTGTTGTACGTAGACGAGGCGTTTGCGTGC
CACTCTGGAACGTTACTTGCTTTGATCGCCTTGGTGAGACCAAGACAGAAAGTTGTACTTTGTGGTGACCC
GAAGCAGTGCGGCTTCTTCAATATGATGCAGATGAAAGTCAACTACAATCATAACATCTGCACCCAAGTGT
ACCACAAAAGTATCTCCAGGCGGTGTACACTGCCTGTGACTGCCATTGTGTCATCGTTGCATTACGAAGGC
AAAATGCGCACTACGAATGAGTACAACATGCCGATTGTAGTGGACACTACAGGCTCAACGAAACCTGACC
CTGGAGACCTCGTGTTAACGTGCTTCAGAGGGTGGGTTAAACAACTGCAAATTGACTATCGTGGACACGA
GGTCATGACAGCAGCCGCATCCCAAGGGTTAACTAGAAAAGGAGTTTACGCAGTTAGGCAAAAAGTTAAC
GAAAACCCACTCTATGCATCAACATCAGAGCACGTCAACGTACTCCTAACGCGTACGGAAGGTAAACTGG
TATGGAAGACACTCTCTGGTGACCCGTGGATAAAGACGCTGCAGAACCCACCGAAAGGAAACTTCAAAGC
AACTATTAAGGAGTGGGAGGTGGAGCACGCATCGATAATGGCGGGCATCTGCAGTCACCAAGTGACCTTT
GACACATTCCAAAACAAAGCCAACGTTTGCTGGGCTAAGAGCTTGGTCCCTATCCTCGAAACAGCGGGGA
TAAAACTAAATGATAGGCAGTGGTCCCAGATAATTCAAGCCTTCAAAGAAGACAAAGCATACTCACCCGA
AGTAGCCCTGAATGAAATATGCACGCGCATGTATGGGGTGGATCTAGACAGTGGGCTATTCTCTAAACCGT
TGGTATCTGTGTATTACGCGGATAACCATTGGGATAATAGGCCGGGAGGAAAGATGTTCGGATTCAACCCT
GAGGCAGCGTCCATTCTAGAAAGAAAGTACCCATTTACAAAAGGAAAGTGGAACATCAACAAGCAGATCT
GCGTGACTACCAGGAGGATAGAAGACTTCAACCCTACCACCAACATTATACCGGTCAACAGGAGACTACC
ACACTCATTAGTGGCCGAACACCGCCCAGTAAAAGGGGAAAGAATGGAATGGCTGGTTAACAAGATAAA
CGGACACCACGTACTCCTGGTTAGCGGCTATAACCTTGCACTGCCTACTAAGAGAGTCACCTGGGTAGCGC
CACTAGGTGTCCGCGGAGCGGACTATACATACAACCTAGAGCTGGGTCTACCAGCAACACTTGGTAGGTA
TGACCTAGTGGTCATAAACATCCACACACCTTTTCGCATACACCATTACCAACAGTGCGTAGATCACGCAA
TGAAACTGCAAATGCTAGGGGGTGACTCACTGAGACTGCTCAAACCGGGTGGCTCTCTATTGATCAGAGC
ATACGGTTACGCAGATAGAACCAGTGAACGAGTCATCTGCGTACTGGGACGCAAGTTTAGATCGTCTAGA
GCATTGAAACCACCATGTGTCACCAGTAATACTGAGATGTTTTTCCTATTTAGCAATTTTGACAATGGCAGA
AGGAATTTTACAACGCATGTCATGAACAATCAACTGAATGCAGCCTTTGTAGGACAGGCCACCCGAGCAG
GATGTGCACCATCGTACCGGGTAAAACGCATGGACATCGCGAAGAACGATGAAGAGTGCGTGGTTAACG
CCGCCAACCCTCGCGGGTTACCAGGTGACGGTGTTTGCAAGGCAGTATATAAAAAGTGGCCGGAGTCCTT
TAAAAACAGTGCAACACCAGTAGGAACCGCAAAAACAGTTATGTGCGGTACGTATCCAGTAATCCACGCC
GTAGGACCAAACTTCTCAAATTATTCGGAGTCTGAAGGGGACCGGGAATTGGCGGCTGCCTATCGAGAAG
TCGCAAAGGAAGTAACTAGACTGGGAGTAAATAGCGTAGCTATACCTCTCCTCTCCACAGGTGTATACTCA
GGAGGGAAAGACAGGCTAACCCAGTCACTGAACCACCTCTTTACAGCCATGGACTCGACGGATGCAGAC
GTGGTCATCTACTGCCGAGACAAGGAATGGGAGAAGAAAATATCTGAGGCCATACAGATGCGGACCCAA
GTGGAGCTGCTGGATGAGCACATCTCCATAGACTGCGATGTCATTCGCGTGCACCCTGACAGTAGCTTGGC
AGGCAGAAAAGGATACAGCACCACGGAAGGCGCACTGTATTCATATCTAGAAGGGACACGTTTTCACCAG
ACGGCAGTGGATATGGCAGAGATATACACTATGTGGCCAAAGCAAACAGAGGCCAATGAGCAAGTCTGC
CTATATGCCCTGGGGGAAAGTATTGAATCAATCAGGCAGAAATGCCCGGTGGATGATGCAGACGCATCAT
CTCCCCCGAAAACTGTCCCGTGTCTTTGCCGGTATGCCATGACTCCTGAACGCGTCACCCGACTTCGCATG

AACCATGTCACAAATATAATTGTGTGTTCTTCATTTCCCCTTCCAAAGTACAAGATAGAAGGAGTGCAAAAA
GTCAAATGCTCCAAGGTAATGTTATTCGATCACAATGTGCCATCGCGCGTAAGTCCAAGGGAATACAGATC
TTCCCAGGAGTCTGTACAGGAAGTGAGTACGACAACGTCATTGACGCATAGCCAGTTTGATCTAAGCGCC
GATGGCGAGACACTGCCTGTCCCGTCAGACCTGGATGCTGACGCCCCAGCCCTAGAACCGGCCCTAGACG
ACGGGGCGGTACATACATTACCAACCATAATCGGAAACCTTGCGGCCGTGTCTGACTGGGTAATGAGCAC
CGTACCTGTCGCGCCGCCTAGAAGAAGGAGAGGGAGAAACCTGACTGTGACATGTGACGAGAGAGAAGG
GAATATAACACCCATGGCTAGCGTCCGATTCTTTAGAGCAGAGCTGTGTCCGGCCGTACAAGAAACAGCG
GAGACGCGTGACACAGCTATTTCCCTTCAGGCACCGCCAAGTACCACCATGGAACTGAGCCATCCACCGA
TCTCCTTCGGAGCACCAAGCGAGACGTTCCCCATCACATTTGGGGACTTCGACGAAGGAGAAATCGAAAG
CTTGTCTTCTGAGCTACTAACTTTCGGAGACTTCCTACCCGGTGAAGTGGATGATCTGACAGATAGCGACT
GGTCCACGTGCCCAGACACGGACGACGAGTTATGACTAGACAGGGCAGGTGGGTATATATTCTCGTCGGA
CACTGGTCCAGGCCATTTACAACAGAAGTCGGTACGCCAGTCAGTGCTGCCGGTAAACACCCTGGAGGAA
GTCCACGAGGAGAAGTGTTACCCACCTAAGCTGGATGAATTAAAGGAGCAACTACTACTTAAGAAACTCC
AGGAGAGTGCGTCCATGGCCAATAGAAGCAGGTATCAGTCACGCAAAGTGGAAAATATGAAAGCAACAA
TCATCCAGAGACTAAAGAGAGGCTGTAAACTGTATTTAATGGCAGAGACCCCGAAAGTCCCGACTTATCG
GACCATATACCCGGCGCCTGTGTACTCGCCTCCGATCAATGTCCGATTGTCCAACCCCGAGTCCGCAGTGG
CAGCATGTAATGAGTTCTTAGCTAGAAACTACCCAACTGTTTCATCATACCAAATCACCGACGAGTATGAT
GCATATCTAGACATGGTGGACGGGTCGGAGAGTTGCTTGGACCGAGCGACATTCAATCCGTCAAAACTTA
GGAGCTACCCGAAACAACATGCTTATCACGCGCCTTCTATCAGAAGCGCTGTACCTTCCCCATTCCAGAAC
ACACTACAGAATGTACTGGCAGCAGCCACGAAAAGGAACTGCAACGTCACACAGATGAGGGAATTACCC
ACTTTGGACTCAGCAGTATTCAACGTGGAGTGTTTTAAAAAATTCGCATGTAACCGAGAATACTGGGAAGA
ATTTGCAGCCAGCCCTATCAGGATAACAACTGAGAATCTAACAACCTATGTCACTAAACTAAAGGGGCCAA
AAGCAGCAGCGCTGTTTGCAAAAACCCATAATCTGCTGCCACTGCAGGATGTACCAATGGATAGGTTCACA
GTAGATATGAAAAGGGATGTGAAGGTAACTCCTGGTACAAAGCATACAGAGGAAAGACCTAAGGTGCAG
GTTATACAGGCGGCTGAACCCTTGGCAACAGCGTACCTATGTGGAATTCACAGAGAACTGGTTAGGAGAT
TGAACGCCGTCCTCCTACCCAATGTGCATACACTATTTGACATGTCTGCCGAGGACTTCGATGCCATTATAG
CCGCACACTTCAAGCCAGGAGACGCTGTTTTAGAAACGGACATAGCCTCCTTTGATAAGAGCCAAGATGAT
TCACTTGCGCTTACCGCCTTAATGCTGTTAGAAGATTTGGGAGTGGATCACTCCCTGTTGGACTTGATAGAG
GCTGCTTTCGGAGAGATTTCCAGCTGTCATCTGCCGACAGGTACGCGCTTCAAGTTCGGCGCTATGATGAA
ATCCGGTATGTTCCTAACTCTGTTCGTCAACACGTTGTTAAATATCACCATCGCTAGCCGGGTGTTGGAAGA
TCGTCTGACAAAATCCGCATGCGCGGCCTTCATCGGCGACGACAACATAATACATGGTGTCGTCTCCGATG
AATTGATGGCAGCCAGATGCGCTACTTGGATGAACATGGAAGTGAAGATCATAGATGCAGTTGTATCCCA
GAAAGCTCCTTACTTTTGTGGAGGGTTTATACTGCATGATACTGTGACAGGAACAGCTTGCAGAGTGGCGG
ACCCGCTAAAAAGGTTATTTAAATTGGGCAAACCGTTAGCGGCAGGTGACGAACAAGATGAAGACAGAAG
ACGGGCGCTGGCTGATGAAGTAATCAGATGGCAACGAACAGGGCTAATAGATGAGCTGGAGAAAGCGGT
GTACTCTAGGTACGAAGTGCAGGGTATATCAGTTGCGGTAATGTCCATGGCCACCTTTGCAAGCTCCAGAT
CCAACTTCGAGAAGCTCAGAGGACCCGTCATAACTTTGTACGGCGGTCCTAAATAGGTACGCACTACAGCT
ACCTATTTTGCAGAAGCCGACAGCAGGTACCTAAATACCAATCAGCCATAATGGAGTTTATCCCAACCCAA
ACTTTCTACAATAGGAGGTACCAGCCTCGACCTTGGACTCCGCGCCCTACTATCCAAGTTATCAGACCCAG
ACCGCGTCCGCAAAGGAAAGCCGGGCAACTTGCCCAGCTGATCTCAGCAGTTAATAAACTGACAATGCGC
GCGGTACCTCAACAGAAGCCGCGCAAGAATCGGAAGAATAAGAAGCAAAAGCAAAAGCAGCAGGCGCC
ACGAAACAACATGAATCAAAAGAAGCAGCCCCCTAAAAAGAAACCGGCTCAAAAGAAAAAGAAGCCGGG
CCGTAGAGAGAGAATGTGCATGAAAATCGAAAATGATTGCATCTTCGAAGTCAAGCATGAAGGTAAGGTA
ACAGGTTACGCGTGCTTGGTAGGGGACAAAGTAATGAAGCCAGCACACGTAAAGGGGACCATCGATAAT
GCGGACCTGGCCAAATTGGCCTTCAAGCGGTCATCTAAGTACGACCTTGAATGCGCGCAGATACCCGTGC

ACATGAAGTCCGACGCTTCGAAGTTCACCCATGAGAAACCGGAGGGGTACTACAACTGGCACCACGGAG
CAGTACAGTACTCAGGAGGCCGGTTCACCATCCCTACAGGTGCGGGCAAACCAGGGGACAGCGGTAGAC
CGATCTTCGACAACAAGGGGCGCGTGGTGGCCATAGTTTTAGGAGGAGCTAATGAAGGAGCCCGTACAGC
CCTCTCGGTGGTGACCTGGAACAAAGACATCGTCACGAAAATCACCCCTGAGGGGGCCGAAGAGTGGAG
TCTTGCCATTatggtgagcgagctgattaaggagaacatgcacatgaagctgtacatggagggcaccgtgaacaaccaccacttcaa
gtgcacatccgagggcgaaggcaagccctacgagggcacccagaccatgagaatcaaggcggtcgagggcggccctctccccttcgcc
ttcgacatcctggctaccagcttcatgtacggcagcaaaaccttcatcaaccacacccagggcatccccgacttctttaagcagtccttccccg
agggcttcacatgggagagagtcaccacatacgaagacgggggcgtgctgaccgctacccaggacaccagcctccaggacggctgcct
catctacaacgtcaagatcagaggggtgaacttcccatccaacggccctgtgatgcagaagaaaacactcggctgggaggcctccaccga
gaccctgtaccccgctgacggcggcctggaaggcagagccgacatggccctgaagctcgtgggcgggggccacctgatctgcaacttg
aagaccacatacagatccaagaaacccgctaagaacctcaagatgcccggcgtctactatgtggacagaagactggaaagaatcaagga
ggccgacaaagagacctacgtcgagcagcacgaggtggctgtggccagatactgcgacctccctagcaaactggggcacagaaactttg
acctacttaagttggcgggagacgttgagtccaaccctgggcccAGTCTTGCCATTCCAGTTATGTGCCTGCTGGCAAATA
CCACGTTCCCCTGCTCCCAGCCCCCTTGCACACCCTGCTGCTACGAAAAAGAGCCGGAGAAAACCCTGCG
CATGCTAGAAGACAACGTCATGAGCCCCGGGTACTATCAGCTGCTACAAGCATCCTTAACATGTTCTCCCC
GCCGCCAGCGACGCAGTATTAAGGACAACTTCAATGTCTATAAAGCCATAAGACCGTACCTAGCTCACTGT
CCCGACTGTGGAGAAGGGCACTCGTGCCATAGTCCCGTAGCGCTAGAACGCATCAGAAACGAAGCGACA
GACGGGACGCTGAAAATCCAGGTTTCCTTGCAAATCGGAATAAAGACGGATGATAGCCATGATTGGACCA
AGCTGCGTTACATGGACAATCATATGCCAGCAGACGCAGAGAGGGCCAGGCTATTTGTAAGAACGTCAGC
ACCGTGCACGATTACTGGAACAATGGGACACTTCATCCTGGCCCGATGTCCGAAAGGAGAAACTCTGACG
GTGGGATTCACTGACGGTAGGAAGATCAGTCACTCATGTACGCACCCATTTCACCACGACCCTCCTGTGAT
AGGCCGGGAAAAATTTCATTCCCGACCGCAGCACGGTAGAGAACTACCTTGCAGCACGTACGCGCAGAGC
ACCGCTGCAACTGCCGAGGAGATAGAGGTACATATGCCCCCAGACACCCCAGATCGCACATTGATGTCAC
AACAGTCCGGTAATGTAAAGATCACAGTCAATAGTCAGACGGTGCGGTACAAGTGTAATTGCGGTGACTC
AAATGAAGGACTAACCACTACAGACAAAGTGATTAATAACTGCAAGGTTGATCAATGCCATGCCGCGGTC
ACCAATCACAAAAAATGGCAGTATAATTCCCCTCTGGTCCCGCGTAATGCTGAACTCGGGGACCGAAAAG
GAAAAGTTCACATTCCGTTTCCTCTGGCAAATGTGACATGCAGGGTGCCTAAGGCAAGGAACCCCACCGT
GACGTACGGAAAAAACCAAGTCATCATGCTGCTGTATCCTGACCACCCAACGCTCCTGTCCTACCGGAATA
TGGGAGAAGAACCAAACTATCAAGAAGAGTGGGTGACGCATAAGAAGGAGATCAGGTTAACCGTGCCGA
CTGAAGGGCTCGAGGTCACGTGGGGCAACAACGAGCCGTACAAGTATTGGCCGCAGTTATCCACAAACG
GTACAGCCCACGGCCACCCGCATGAGATAATTTTGTATTATTATGAGCTGTACCCTACTATGACTGTGGTAG
TTGTGTCAGTGGCCTCGTTCGTACTCCTGTCGATGGTGGGTGTGGCAGTGGGGATGTGCATGTGTGCACGA
CGCAGATGCATTACACCGTACGAACTGACACCAGGAGCTACCGTCCCTTTCCTGCTTAGCCTAATATGCTG
CATTAGAACAGCTAAAGCGGCCACATACCAAGAGGCTGCGGTATACCTGTGGAACGAGCAGCAGCCTTTG
TTTTGGCTGCAAGCCCTTATTCCGCTGGCAGCCCTGATTGTCCTATGCAACTGTCTGAGACTCTTACCATGC
TTTTGTAAAACGTTGACTTTTTTAGCCGTAATGAGCGTCGGTGCCCACACTGTGAGCGCGTACGAACACGT
AACAGTGATCCCGAACACGGTGGGAGTACCGTATAAGACTCTAGTCAACAGACCGGGCTACAGCCCCATG
GTACTGGAGATGGAGCTTCTGTCAGTCACTTTGGAGCCAACGCTATCGCTTGATTACATCACGTGCGAGTA
TAAAACCGTCATCCCGTCTCCGTACGTGAAATGCTGCGGTACAGCAGAGTGCAAGGACAAGAGCCTACCT
GATTACAGCTGTAAGGTCTTCACCGGCGTCTACCCATTCATGTGGGGCGGCGCCTACTGCTTCTGCGACAC
TGAAAATACGCAATTGAGCGAAGCACATGTGGAGAAGTCCGAATCATGCAAAACAGAATTTGCATCAGCA
TATAGGGCTCATACCGCATCCGCATCAGCTAAGCTCCGCGTCCTTTACCAAGGAAATAATGTTACTGTATCT
GCTTATGCAAACGGCGATCATGCCGTCACAGTTAAGGACGCTAAATTCATTGTGGGGCCAATGTCTTCAGC
CTGGACACCTTTTGACAATAAAATCGTGGTGTACAAAGGCGACGTCTACAACATGGACTACCCGCCCTTCG
GCGCAGGAAGACCAGGACAATTTGGCGACATCCAAAGTCGCACGCCTGAGAGCGAAGACGTCTATGCTA

ACACACAACTGGTACTGCAGAGACCGTCCGCGGGTACGGTGCACGTGCCGTACTCTCAGGCACCATCTGG
CTTCAAGTATTGGCTAAAAGAACGAGGGGCGTCGCTGCAGCACACAGCACCATTTGGCTGTCAAATAGCA
ACAAACCCGGTAAGAGCGATGAACTGCGCCGTAGGGAACATGCCTATCTCCATCGACATACCGGACGCGG
CCTTCACTAGGGTCGTCGACGCGCCATCTTTAACGGACATGTCGTGTGAGGTACCAGCCTGCACCCACTCC
TCAGACTTTGGGGGCGTAGCCATCATTAAATATGCAGCCAGCAAGAAAGGCAAGTGTGCGGTGCATTCGA
TGACTAACGCCGTCACTATTCGGGAAGCTGAAATAGAAGTAGAAGGGAACTCTCAGTTGCAAATCTCTTTT
TCGACGGCCCTAGCCAGCGCCGAATTCCGCGTACAAGTCTGTTCTACACAAGTACACTGTGCAGCCGAGT
GCCATCCACCGAAAGACCATATAGTCAATTACCCGGCGTCACACACCACCCTCGGGGTCCAAGACATTTCC
GTTACGGCGATGTCATGGGTGCAGAAGATCACGGGAGGTGTGGGACTGGTTGTCGCTGTTGCAGCACTGA
TCCTAATCGTGGTGCTATGCGTGTCGTTTAGCAGGCACTAACTTGACAACTAGGTACGAAGGTATATGTGT
CCCCTAAGAGACACACCACATATAGCTAAGAATCAATAGATAAGTATAGATCAAAGGGCTGAACAACCCC
TGAATAGTAACAAAATATAAAAATCAACAAAAATCATAAAATAGAAAACCAGAAACAGAAGTAGGTAAGA
AGGTATATGTGTCCCCTAAGAGACACACCATATATAGCTAAGAATCAATAGATAAGTATAGATCAAAGGGC
TGAATAACCCCTGAATAATAACAAAATATAAAAATCAATAAAAATCATAAAATAGAAAACCATAAACAGAA
GTAGTTCAAAGGGCTATAAAACCCCTGAAAAGTAACAAAACATAAAACTAATAAAAATCAAATGAATACC
ATAATTGGCAATCGGAAGAGATGTAGGTACTTAAGCTTCCTAAAAGCAGCCGAACTCGCTTTGAGATGTAG
GCGTAGCACACCGAACTCTTCCATAATTCTCCGAACCCACAGGGACGTAGGAGATGTTCAAAGTGGCTATA
AAACCCTGAACAGTAATAAAACATAAAATTAATAAGGATCAAATGAGTACCATAATTGGCAAACGGAAGA
GATGTAGGTACTTAAGCTTCCTAAAAGCAGCCGAACTCACTTTGAGATGTAGGCATAGCATACCGAACTCT
TCCACAATTCTCCGTACCCATAGGGACGTAGGAGATGTTATTTTGTTTTTAATATTTCAAAAAAAAAAAAAA
AAAAAAAAAAAAAgggtcggcatggcatctccacctcctcgcggtccgacctgggcatccgaaggaggacgcacgtccactcggat
ggctaagggagagccacgagctcctcgacagatcataatcagccataccacatttgtagaggttttacttgctttaaaaaacctcccacacctc
cccctgaacctgaaacataaaatgaatgcaattgttgttgttaacttgtttattgcagcttataatggttacaaataaagcaatagcatcacaaat
ttcacaaataaagcatttttttcactgcattctagttgtggtttgtccaaactcatcaagataataaaggaaatttattttcattgcaatagtgtgtt
ggaatttttgtgtctctcactcggaaggacatatgggagggcaaatcatttaaaacatcagaatgagtatttggtttagagtttggcaacata
tgcccatatgtaagatatcaggcttcgagcaagacgtttcccgttgaatatggctcataacaccccttgtattactgtttatgtaagcagacagt
tttattgttcatgatgatatatttttatcttgtgcaatgtaacatcagagattttgagacacaacgtggctttgttgaataaatcgaacttttgctga
gttgaaggatcagatcacgcatcttcccgacaacgcagaccgttccgtggcaaagcaaaagttcaaaatcaccaactggtccacctacaaca
aagctctcatcaaccgtggctccctcactttctggctggatgatggggcgattcaggcctggtatgagtcagcaacaccttcttcacgaggca
gacctcagcgctagcggagtgtatactggcttactatgttggcactgatgagggtgtcagtgaagtgcttcatgtggcaggagaaaaaag
gctgcaccggtgcgtcagcagaatatgtgatacaggatatattccgcttcctcgctcactgactcgctacgctcggtcgttcgactgcggcga
gcggaaatggcttacgaacggggcggagatttcctggaagatgccaggaagatacttaacagggaagtgagagggccgcggcaaagc
cgtttttccataggctccgcccccctgacaagcatcacgaaatctgacgctcaaatcagtggtggcgaaacccgacaggactataaagatac
caggcgtttccccctggcggctccctcgtgcgctctcctgttcctgcctttcggtttaccggtgtcattccgctgttatggccgcgtttgtctcattc
cacgcctgacactcagttccgggtaggcagttcgctccaagctggactgtatgcacgaaccccccgttcagtccgaccgctgcgccttatcc
ggtaactatcgtcttgagtccaacccggaaagacatgcaaaagcaccactggcagcagccactggtaattgatttagaggagttagtcttga
agtcatgcgccggttaaggctaaactgaaaggacaagttttggtgactgcgctcctccaagccagttacctcggttcaaagagttggtagct
cagagaaccttcgaaaaaccgccctgcaaggcggttttttcgttttcagagcaagagattacgcgcagaccaaaacgatctcaagaagatc
atcttattaaggggtctgacgctcagtggaacgaaaactcacgttaagggattttggtcatgagattatcaaaaaggatcttcacctagatcct
tttaaattaaaaatgaagttttaaatcaatctaaagtatatatgagtaaacttggtctgacagttaccaatgcttaatcagtgaggcacctatctc
agcgatctgtctatttcgttcatccatagttgcctgactccccgtcgtgtagataactacgatacgggagggcttaccatctggccccagtgct
gcaatgataccgcgagacccacgctcaccggctccagatttatcagcaataaaccagccagccggaagggccgagcgcagaagtggtcc
tgcaactttatccgcctccatccagtctattaattgttgccgggaagctagagtaagtagttcgccagttaatagtttgcgcaacgttgttgcca
ttgctgcaggcatcgtggtgtcacgctcgtcgtttggtatggcttcattcagctccggttcccaacgatcaaggcgagttacatgatcccccat
gttgtgcaaaaaagcggttagctccttcggtcctccgatcgttgtcagaagtaagttggccgcagtgttatcactcatggttatggcagcact

gcataattctcttactgtcatgccatccgtaagatgcttttctgtgactggtgagtactcaaccaagtcattctgagaatagtgtatgcggcgac
cgagttgctcttgcccggcgtcaacacgggataataccgcgccacatagcagaactttaaaagtgctcatcattggaaaacgttcttcgggg
cgaaaactctcaaggatcttaccgctgttgagatccagttcgatgtaacccactcgtgcacccaactgatcttcagcatcttttactttcaccagc
gtttctgggtgagcaaaaacaggaaggcaaaatgccgcaaaaaagggaataagggcgacacggaaatgttgaatactcatactcttccttt
ttcaatattattgaagcatttatcagggttattgtctcatgagcggatacatatttgaatgtatttagaaaaataaacaaataggggttccgcgc
acatttccccgaaaagtgccacctgacgtgtcgacgcggccgcatgtcgttacataacttacggtaaatggcccgcctggctgaccgcccaa
cgacccccgcccattgacgtcaataatgacgtatgttcccatagtaacgccaatagggactttccattgacgtcaatgggtggagtatttacg
gtaaactgcccacttggcagtacatcaagtgtatcatatgccaagtacgccccctattgacgtcaatgacggtaaatggcccgcctggcatta
tgcccagtacatgaccttatgggactttcctacttggcagtacatctacgtattagtcatcgctattaccatggtgatgcggttttggcagtacat
caatgggcgtggatagcggtttgactcacggggatttccaagtctccaccccattgacgtcaatgggagtttgttttggcaccaaaatcaac
gggactttccaaaatgtcgtaacaactccgccccattgacgcaaatgggcggtaggcgtgtacggtgggaggtctatataagcagagctc
gtttagtgaaccgt

pACNR-CHIKV plasmid sequence annotated

Feature	Location (nts)	Orientation with respect the CMV promoter	Type
5′ UTR	1 to 76	Right	5′ Untranslated region
nsp1	77 to 1,681	Right	CDS – nonstructural protein 1 – 59.5 kDa
nsp2	1,682 to 4,075	Right	CDS – nonstructural protein 2 – 89.6 kDa
nsp3	4,076 to 5,665	Right	CDS – nonstructural protein 3 – 57.5 kDa
nsp4	5,666 to 7,501	Right	CDS – nonstructural protein 3 – 68.2 kDa
SG promoter	7,472 to 7,504	Right	Promoter for the subgenomic RNA
Capsid	7,567 to 8,394	Right	CDS – capsid protein – 29.6 kDa
E3 (partial)	8,350 to 8,361	Right	CDS – partial sequence of the E3 chaperone – This allows the correct autoproteolytic cleavage between C and mKate2 – 402 Da
mKate2	8,362 to 9,057	Right	CDS – reporter gene mKate2 – 26.1 kDa
FMV2	9,058 to 9,108	Right	CDS – Foot and mouse disease virus cleave site to separate mKate 2 from E3.
E3	9,109 to 9,300	Right	CDS – E3 protein – 7.3 kDa
E2	9301 to 10,569	Right	CDS – E2 glycoprotein – 47.5 kDa
6K	10,570 to 10,752	Right	CDS – 6K protein – 6.8 kDa

E1	10,752 to 12,072	Right	CDS – E1glycoprotein – 47.5 kDa
3′ UTR	12,073 to 12,822	Right	3′ Untranslated region that includes the viral polyA
HDV	12,823 to 12,906	Right	Hepatitis delta virus ribozyme
SV40 polyA	13,032 to 13,147	Right	Simian vacuolating virus 40 polyadenylation signal and transcription terminator
β-globin polyA	13,148 to 13,203	Right	β-globin polyadenylation signal and transcription terminator
p15A ori	13,930 to 14,474	Left	Origin of replication for *E. coli*
AmpR	14,639 to 15,499	Left	Ampicillin resistant gene for selection in *E. coli* – 31.5 kDa
AmpR promoter	15,500 to 15,604	Left	*E. coli* promoter for AmpR
CMV enhancer	15,653 to 15,956	Right	Enhancer for the Cytomegalovirus promoter for mammalian cell lines.
CMV promoter	15,957 to 16,169	Right	Cytomegalovirus transcription promoter for mammalian cell lines.

pACNR-CHIKV plasmid map

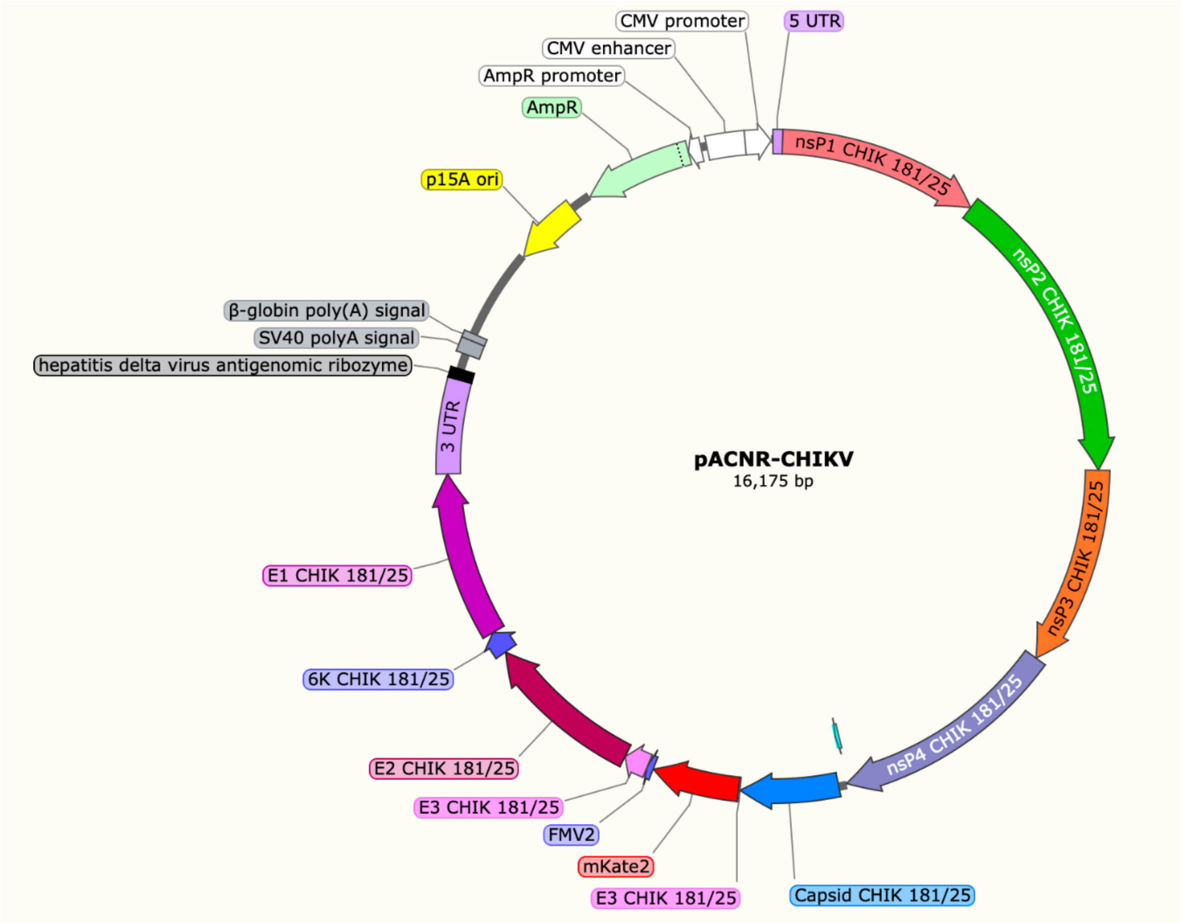

pACNR-Rep plasmid sequence

ATGGCTGCGTGAGACACACGTAGCCTACCAGTTTCTTACTGCTCTACTCTGCAAAGCAAGAGATTAATAAC
CCATCATGGATTCTGTGTACGTGGACATAGACGCTGACAGCGCCTTTTTGAAGGCCCTGCAACGTGCGTAC
CCCATGTTTGAGGTGGAACCTAGGCAGGTCACATCGAATGACCATGCTAATGCTAGAGCGTTCTCGCATCT
AGCCATAAAACTAATAGAGCAGGAAATTGATCCCGACTCAACCATCCTGGATATAGGTAGTGCGCCAGCA
AGGAGGATGATGTCGGACAGGAAGTACCACTGCGTTTGCCCGATGCGCAGCGCAGAAGATCCCGAGAGA
CTCGCTAATTATGCGAGAAAGCTCGCATCTGCCGCAGGAAAAGTCCTGGACAGAAACATTTCTGGAAAGA
TCGGGGACTTACAAGCGGTGATGGCCGTGCCAGACACGGAGACGCCAACATTTTGCTTACACACAGATGT
CTCATGTAGACAGAGAGCAGACGTCGCGATATACCAAGACGTCTATGCTGTACACGCACCCACGTCGCTA
TACCACCAGGCGATTAAAGGAGTCCGAGTGGCGTACTGGGTAGGGTTCGACACAACCCCGTTCATGTACA
ACGCTATGGCGGGTGCCTACCCCTCATACTCGACAAATTGGGCGGATGAGCAGGTACTGAAGGCTAAGAA
CATAGGATTATGTTCAACAGACCTGACGGAAGGTAGACGAGGCAAATTGTCTATCATGAGAGGGAAAAAG
CTAAAACCGTGCGACCGTGTGCTGTTCTCAGTAGGGTCAACGCTTTACCCGGAAAGCCGCACGCTACTTAA
GAGCTGGCACCTACCATCGGTGTTCCATCTAAAGGGCAAGCTTAGCTTCACATGCCGCTGTGACACAGTGG
TTTCGTGTGAGGGCTACGTCGTTAAGAGAATAACGATGAGCCCAGGCCTTTATGGAAAAACCATAGGGTAT
GCGGTAACCCACCACGCAGACGGATTCTTGATGTGCAAGACTACCGACACGGTTGACGGCGAAAGAGTGT
CATTCTCGGTGTGCACGTACGTGCCGGCGACCATTTGTGATCAAATGACCGGCATCCTTGCTACAGAAGTC
ACGCCGGAGGATGCACAGAAGCTGTTGGTGGGGCTGAACCAGAGGATAGTGGTTAACGGCAGAACGCAA
CGGAACACGAACACCATGAAGAACTACCTACTTCCCGTGGTCGCCCAGGCCTTCAGTAAGTGGGCAAAGG

AGTGCCGGAAGGACATGGAAGATGAGAAGCTTCTGGGGGTCAGAGAAAGAACACTAACCTGCTGCTGTCT
ATGGGCATTTAAGAAGCAGAAAACACACACGGTCTACAAGAGGCCTGATACCCAGTCAATCCAGAAGGTT
CAGGCCGAATTTGACAGCTTTGTAGTACCGGGCCTGTGGTCGTCCGGGTTGTCAATCCCGTTGAGGACTAG
AATCAAGTGGTTGTTACGCAAGGTGCCGAAAACAGACCTGATCCCATACAGCGGGAATGCCCAAGAAGCC
CAGGATGCAGAAAAAGAAGCAGAGGAAGAACGAGAAGCAGAACTGACTCATGAGGCTCTACCACCCCTA
CAGGCAGCACAGGAAGATGTCCAGGTCGAAATCGACGTGGAACAGCTTGAGGATAGAGCTGGTGCTGGA
ATAATAGAGACTCCGAGAGGCGCTATCAAAGTTACTGCCCAACTAACAGACCACGTCGTGGGGGAGTACC
TGGTACTTTCCCCGCAGACCGTACTACGCAGCCAGAAGCTCAGCCTGATCCACGCTTTAGCGGAGCAAGT
GAAGACGTGTACGCACAGCGGACGAGCAGGGAGGTATGCGGTCGAAGCGTACGATGGCCGAGTCCTAGT
GCCCTCAGGCTATGCAATTTCGCCTGAAGACTTCCAGAGTCTAAGCGAAAGCGCAACGATGGTGTACAAC
GAAAGAGAGTTCGTAAACAGAAAGTTACACCACATTGCGATGCACGGACCAGCCCTGAACACTGACGAA
GAGTCGTATGAGCTGGTGAGGGCAGAGAGGACAGAACACGAGTACGTCTACGACGTGGACCAGAGAAGA
TGCTGTAAGAAGGAAGAAGCTGCAGGACTGGTACTGGTGGGCGACTTGACTAATCCGCCCTACCACGAAT
TCGCATACGAAGGGCTAAAAATTCGCCCCGCCTGCCCATACAAAATTGCAGTCATAGGAGTCTTCGGGGTA
CCAGGATCTGGCAAGTCAGCCATTATCAAGAACCTAGTTACCAGGCAAGACCTGGTGACTAGCGGAAAGA
AAGAAAACTGCCAAGAAATCAGCACCGACGTGATGAGACAGAGAGGTCTAGAGATATCTGCACGTACGG
TAGATTCGCTGCTCTTGAATGGATGCAACAGACCAGTCGACGTGTTGTACGTAGACGAGGCGTTTGCGTGC
CACTCTGGAACGTTACTTGCTTTGATCGCCTTGGTGAGACCAAGACAGAAAGTTGTACTTTGTGGTGACCC
GAAGCAGTGCGGCTTCTTCAATATGATGCAGATGAAAGTCAACTACAATCATAACATCTGCACCCAAGTGT
ACCACAAAAGTATCTCCAGGCGGTGTACACTGCCTGTGACTGCCATTGTGTCATCGTTGCATTACGAAGGC
AAAATGCGCACTACGAATGAGTACAACATGCCGATTGTAGTGGACACTACAGGCTCAACGAAACCTGACC
CTGGAGACCTCGTGTTAACGTGCTTCAGAGGGTGGGTTAAACAACTGCAAATTGACTATCGTGGACACGA
GGTCATGACAGCAGCCGCATCCCAAGGGTTAACTAGAAAAGGAGTTTACGCAGTTAGGCAAAAAGTTAAC
GAAAACCCACTCTATGCATCAACATCAGAGCACGTCAACGTACTCCTAACGCGTACGGAAGGTAAACTGG
TATGGAAGACACTCTCTGGTGACCCGTGGATAAAGACGCTGCAGAACCCACCGAAAGGAAACTTCAAAGC
AACTATTAAGGAGTGGGAGGTGGAGCACGCATCGATAATGGCGGGCATCTGCAGTCACCAAGTGACCTTT
GACACATTCCAAAACAAAGCCAACGTTTGCTGGGCTAAGAGCTTGGTCCCTATCCTCGAAACAGCGGGGA
TAAAACTAAATGATAGGCAGTGGTCCCAGATAATTCAAGCCTTCAAAGAAGACAAAGCATACTCACCCGA
AGTAGCCCTGAATGAAATATGCACGCGCATGTATGGGGTGGATCTAGACAGTGGGCTATTCTCTAAACCGT
TGGTATCTGTGTATTACGCGGATAACCATTGGGATAATAGGCCGGGAGGAAAGATGTTCGGATTCAACCCT
GAGGCAGCGTCCATTCTAGAAAGAAAGTACCCATTTACAAAAGGAAAGTGGAACATCAACAAGCAGATCT
GCGTGACTACCAGGAGGATAGAAGACTTCAACCCTACCACCAACATTATACCGGTCAACAGGAGACTACC
ACACTCATTAGTGGCCGAACACCGCCCAGTAAAAGGGGAAAGAATGGAATGGCTGGTTAACAAGATAAA
CGGACACCACGTACTCCTGGTTAGCGGCTATAACCTTGCACTGCCTACTAAGAGAGTCACCTGGGTAGCGC
CACTAGGTGTCCGCGGAGCGGACTATACATACAACCTAGAGCTGGGTCTACCAGCAACACTTGGTAGGTA
TGACCTAGTGGTCATAAACATCCACACACCTTTTCGCATACACCATTACCAACAGTGCGTAGATCACGCAA
TGAAACTGCAAATGCTAGGGGGTGACTCACTGAGACTGCTCAAACCGGGTGGCTCTCTATTGATCAGAGC
ATACGGTTACGCAGATAGAACCAGTGAACGAGTCATCTGCGTACTGGGACGCAAGTTTAGATCGTCTAGA
GCATTGAAACCACCATGTGTCACCAGTAATACTGAGATGTTTTTCCTATTTAGCAATTTTGACAATGGCAGA
AGGAATTTTACAACGCATGTCATGAACAATCAACTGAATGCAGCCTTTGTAGGACAGGCCACCCGAGCAG
GATGTGCACCATCGTACCGGGTAAAACGCATGGACATCGCGAAGAACGATGAAGAGTGCGTGGTTAACG
CCGCCAACCCTCGCGGGTTACCAGGTGACGGTGTTTGCAAGGCAGTATATAAAAAGTGGCCGGAGTCCTT
TAAAAACAGTGCAACACCAGTAGGAACCGCAAAAACAGTTATGTGCGGTACGTATCCAGTAATCCACGCC
GTAGGACCAAACTTCTCAAATTATTCGGAGTCTGAAGGGGACCGGGAATTGGCGGCTGCCTATCGAGAAG
TCGCAAAGGAAGTAACTAGACTGGGAGTAAATAGCGTAGCTATACCTCTCCTCTCCACAGGTGTATACTCA

GGAGGGAAAGACAGGCTAACCCAGTCACTGAACCACCTCTTTACAGCCATGGACTCGACGGATGCAGAC
GTGGTCATCTACTGCCGAGACAAGGAATGGGAGAAGAAAATATCTGAGGCCATACAGATGCGGACCCAA
GTGGAGCTGCTGGATGAGCACATCTCCATAGACTGCGATGTCATTCGCGTGCACCCTGACAGTAGCTTGGC
AGGCAGAAAAGGATACAGCACCACGGAAGGCGCACTGTATTCATATCTAGAAGGGACACGTTTTCACCAG
ACGGCAGTGGATATGGCAGAGATATACACTATGTGGCCAAAGCAAACAGAGGCCAATGAGCAAGTCTGC
CTATATGCCCTGGGGGAAAGTATTGAATCAATCAGGCAGAAATGCCCGGTGGATGATGCAGACGCATCAT
CTCCCCCGAAAACTGTCCCGTGTCTTTGCCGGTATGCCATGACTCCTGAACGCGTCACCCGACTTCGCATG
AACCATGTCACAAATATAATTGTGTGTTCTTCATTTCCCCTTCCAAAGTACAAGATAGAAGGAGTGCAAAAA
GTCAAATGCTCCAAGGTAATGTTATTCGATCACAATGTGCCATCGCGCGTAAGTCCAAGGGAATACAGATC
TTCCCAGGAGTCTGTACAGGAAGTGAGTACGACAACGTCATTGACGCATAGCCAGTTTGATCTAAGCGCC
GATGGCGAGACACTGCCTGTCCCGTCAGACCTGGATGCTGACGCCCCAGCCCTAGAACCGGCCCTAGACG
ACGGGGCGGTACATACATTACCAACCATAATCGGAAACCTTGCGGCCGTGTCTGACTGGGTAATGAGCAC
CGTACCTGTCGCGCCGCCTAGAAGAAGGAGAGGGAGAAACCTGACTGTGACATGTGACGAGAGAGAAGG
GAATATAACACCCATGGCTAGCGTCCGATTCTTTAGAGCAGAGCTGTGTCCGGCCGTACAAGAAACAGCG
GAGACGCGTGACACAGCTATTTCCCTTCAGGCACCGCCAAGTACCACCATGGAACTGAGCCATCCACCGA
TCTCCTTCGGAGCACCAAGCGAGACGTTCCCCATCACATTTGGGGACTTCGACGAAGGAGAAATCGAAAG
CTTGTCTTCTGAGCTACTAACTTTCGGAGACTTCCTACCCGGTGAAGTGGATGATCTGACAGATAGCGACT
GGTCCACGTGCCCAGACACGGACGACGAGTTATGACTAGACAGGGCAGGTGGGTATATATTCTCGTCGGA
CACTGGTCCAGGCCATTTACAACAGAAGTCGGTACGCCAGTCAGTGCTGCCGGTAAACACCCTGGAGGAA
GTCCACGAGGAGAAGTGTTACCCACCTAAGCTGGATGAATTAAAGGAGCAACTACTACTTAAGAAACTCC
AGGAGAGTGCGTCCATGGCCAATAGAAGCAGGTATCAGTCACGCAAAGTGGAAAATATGAAAGCAACAA
TCATCCAGAGACTAAAGAGAGGCTGTAAACTGTATTTAATGGCAGAGACCCCGAAAGTCCCGACTTATCG
GACCATATACCCGGCGCCTGTGTACTCGCCTCCGATCAATGTCCGATTGTCCAACCCCGAGTCCGCAGTGG
CAGCATGTAATGAGTTCTTAGCTAGAAACTACCCAACTGTTTCATCATACCAAATCACCGACGAGTATGAT
GCATATCTAGACATGGTGGACGGGTCGGAGAGTTGCTTGGACCGAGCGACATTCAATCCGTCAAAACTTA
GGAGCTACCCGAAACAACATGCTTATCACGCGCCTTCTATCAGAAGCGCTGTACCTTCCCCATTCCAGAAC
ACACTACAGAATGTACTGGCAGCAGCCACGAAAAGGAACTGCAACGTCACACAGATGAGGGAATTACCC
ACTTTGGACTCAGCAGTATTCAACGTGGAGTGTTTTAAAAAATTCGCATGTAACCGAGAATACTGGGAAGA
ATTTGCAGCCAGCCCTATCAGGATAACAACTGAGAATCTAACAACCTATGTCACTAAACTAAAGGGGCCAA
AAGCAGCAGCGCTGTTTGCAAAAACCCATAATCTGCTGCCACTGCAGGATGTACCAATGGATAGGTTCACA
GTAGATATGAAAAGGGATGTGAAGGTAACTCCTGGTACAAAGCATACAGAGGAAAGACCTAAGGTGCAG
GTTATACAGGCGGCTGAACCCTTGGCAACAGCGTACCTATGTGGAATTCACAGAGAACTGGTTAGGAGAT
TGAACGCCGTCCTCCTACCCAATGTGCATACACTATTTGACATGTCTGCCGAGGACTTCGATGCCATTATAG
CCGCACACTTCAAGCCAGGAGACGCTGTTTTAGAAACGGACATAGCCTCCTTTGATAAGAGCCAAGATGAT
TCACTTGCGCTTACCGCCTTAATGCTGTTAGAAGATTTGGGAGTGGATCACTCCCTGTTGGACTTGATAGAG
GCTGCTTTCGGAGAGATTTCCAGCTGTCATCTGCCGACAGGTACGCGCTTCAAGTTCGGCGCTATGATGAA
ATCCGGTATGTTCCTAACTCTGTTCGTCAACACGTTGTTAAATATCACCATCGCTAGCCGGGTGTTGGAAGA
TCGTCTGACAAAATCCGCATGCGCGGCCTTCATCGGCGACGACAACATAATACATGGTGTCGTCTCCGATG
AATTGATGGCAGCCAGATGCGCTACTTGGATGAACATGGAAGTGAAGATCATAGATGCAGTTGTATCCCA
GAAAGCTCCTTACTTTTGTGGAGGGTTTATACTGCATGATACTGTGACAGGAACAGCTTGCAGAGTGGCGG
ACCCGCTAAAAAGGTTATTTAAATTGGGCAAACCGTTAGCGGCAGGTGACGAACAAGATGAAGACAGAAG
ACGGGCGCTGGCTGATGAAGTAATCAGATGGCAACGAACAGGGCTAATAGATGAGCTGGAGAAAGCGGT
GTACTCTAGGTACGAAGTGCAGGGTATATCAGTTGCGGTAATGTCCATGGCCACCTTTGCAAGCTCCAGAT
CCAACTTCGAGAAGCTCAGAGGACCCGTCATAACTTTGTACGGCGGTCCTAAATAGGTACGCACTACAGCT
ACCTATTTTGCAGAAGCCGACAGCAGGTACCTAAATACCAATCAGCCATAATGGAGGCCATTATGGTGAG

CGAGctgattaaggagaacatgcacatgaagctgtacatggagggcaccgtgaacaaccaccacttcaagtgcacatccgagggcgaa
ggcaagccctacgagggcacccagaccatgagaatcaaggcggtcgagggcggccctctcccttcgccttcgacatcctggctaccagc
ttcatgtacggcagcaaaaccttcatcaaccacacccagggcatccccgacttctttaagcagtccttccccgagggcttcacatgggagaga
gtcaccacatacgaagacgggggcgtgctgaccgctacccaggacaccagcctccaggacggctgcctcatctacaacgtcaagatcaga
ggggtgaacttcccatccaacggccctgtgatgcagaagaaaacactcggctgggaggcctccaccgagaccctgtaccccgctgacgg
cggcctggaaggcagagccgacatggccctgaagctcgtgggcgggggccacctgatctgcaacttgaagaccacatacagatccaag
aaacccgctaagaacctcaagatgcccggcgtctactatgtggacagaagactggaaagaatcaaggaggccgacaaagagacctacgt
cgagcagcacgaggtggctgtggccagatactgcgacctcccTAGCAAACTGGGGCACAGACTTGACAACTAGGTACGA
AGGTATATGTGTCCCCTAAGAGACACACCACATATAGCTAAGAATCAATAGATAAGTATAGATCAAAGGG
CTGAACAACCCCTGAATAGTAACAAAATATAAAAATCAACAAAAATCATAAAATAGAAAACCAGAAACAG
AAGTAGGTAAGAAGGTATATGTGTCCCCTAAGAGACACCATATATAGCTAAGAATCAATAGATAAGTAT
AGATCAAAGGGCTGAATAACCCCTGAATAATAACAAAATATAAAAATCAATAAAAATCATAAAATAGAAA
ACCATAAACAGAAGTAGTTCAAAGGGCTATAAAACCCCTGAAAAGTAACAAAACATAAAACTAATAAAAA
TCAAATGAATACCATAATTGGCAATCGGAAGAGATGTAGGTACTTAAGCTTCCTAAAAGCAGCCGAACTCG
CTTTGAGATGTAGGCGTAGCACACCGAACTCTTCCATAATTCTCCGAACCCACAGGGACGTAGGAGATGTT
CAAAGTGGCTATAAAACCCTGAACAGTAATAAAACATAAAATTAATAAGGATCAAATGAGTACCATAATTG
GCAAACGGAAGAGATGTAGGTACTTAAGCTTCCTAAAAGCAGCCGAACTCACTTTGAGATGTAGGCATAG
CATACCGAACTCTTCCACAATTCTCCGTACCCATAGGGACGTAGGAGATGTTATTTTGTTTTTAATATTTCAA
AAAAAAAAAAAAAAAAAAAAAAAAAgggtcggcatggcatctccacctcctcgcggtccgacctgggcatccgaaggaggac
gcacgtccactcggatggctaagggagagccacgagctcctcgacagatcataatcagccataccacatttgtagaggttttacttgctttaa
aaaacctcccacacctccccctgaacctgaaacataaaatgaatgcaattgttgttgttaacttgtttattgcagcttataatggttacaaataaa
gcaatagcatcacaaatttcacaaataaagcatttttttcactgcattctagttgtggtttgtccaaactcatcaagataataaaggaaatttatttt
cattgcaatagtgtgttggaattttttgtgtctctcactcggaaggacatatgggagggcaaatcatttaaaacatcagaatgagtatttggttt
agagtttggcaacatatgcccatatgtaagatatcaggcttcgagcaagacgtttcccgttgaatatggctcataacaccccttgtattactgtt
tatgtaagcagacagttttattgttcatgatgatatatttttatcttgtgcaatgtaacatcagagattttgagacacaacgtggctttgttgaata
aatcgaacttttgctgagttgaaggatcagatcacgcatcttcccgacaacgcagaccgttccgtggcaaagcaaaagttcaaaatcaccaa
ctggtccacctacaacaaagctctcatcaaccgtggctccctcactttctggctggatgatggggcgattcaggcctggtatgagtcagcaac
accttcttcacgaggcagacctcagcgctagcggagtgtatactggcttactatgttggcactgatgagggtgtcagtgaagtgcttcatgt
ggcaggagaaaaaaggctgcaccggtgcgtcagcagaatatgtgatacaggatatattccgcttcctcgctcactgactcgctacgctcgg
tcgttcgactgcggcgagcggaaatggcttacgaacggggcggagatttcctggaagatgccaggaagatacttaacagggaagtgag
agggccgcggcaaagccgtttttccataggctccgcccccctgacaagcatcacgaaatctgacgctcaaatcagtggtggcgaaacccga
caggactataaagataccaggcgtttccccctggcggctccctcgtgcgctctcctgttcctgcctttcggtttaccggtgtcattccgctgttatg
gccgcgtttgtctcattccacgcctgacactcagttccgggtaggcagttcgctccaagctggactgtatgcacgaaccccccgttcagtccg
accgctgcgccttatccggtaactatcgtcttgagtccaacccggaaagacatgcaaaagcaccactggcagcagccactggtaattgattt
agaggagttagtcttgaagtcatgcgccggttaaggctaaactgaaaggacaagttttggtgactgcgctcctccaagccagttacctcggt
tcaaagagttggtagctcagagaaccttcgaaaaaccgccctgcaaggcggttttttcgttttcagagcaagagattacgcgcagaccaaaa
cgatctcaagaagatcatcttattaaggggtctgacgctcagtggaacgaaaactcacgttaagggattttggtcatgagattatcaaaaag
gatcttcacctagatccttttaaattaaaaatgaagttttaaatcaatctaaagtatatatgagtaaacttggtctgacagttaccaatgcttaatc
agtgaggcacctatctcagcgatctgtctatttcgttcatccatagttgcctgactccccgtcgtgtagataactacgatacgggagggcttac
catctggccccagtgctgcaatgataccgcgagacccacgctcaccggctccagatttatcagcaataaaccagccagccggaagggccg
agcgcagaagtggtcctgcaactttatccgcctccatccagtctattaattgttgccgggaagctagagtaagtagttcgccagttaatagttt
gcgcaacgttgttgccattgctgcaggcatcgtggtgtcacgctcgtcgtttggtatggcttcattcagctccggttcccaacgatcaaggcg
agttacatgatcccccatgttgtgcaaaaaagcggttagctccttcggtcctccgatcgttgtcagaagtaagttggccgcagtgttatcactc
atggttatggcagcactgcataattctcttactgtcatgccatccgtaagatgcttttctgtgactggtgagtactcaaccaagtcattctgaga
atagtgtatgcggcgaccgagttgctcttgcccggcgtcaacacgggataataccgcgccacatagcagaactttaaaagtgctcatcattg

gaaaacgttcttcggggcgaaaactctcaaggatcttaccgctgttgagatccagttcgatgtaacccactcgtgcacccaactgatcttcag
catcttttactttcaccagcgtttctgggtgagcaaaaacaggaaggcaaaatgccgcaaaaaagggaataagggcgacacggaaatgtt
gaatactcatactcttcctttttcaatattattgaagcatttatcagggttattgtctcatgagcggatacatatttgaatgtatttagaaaaataaa
caaataggggttccgcgcacatttccccgaaaagtgccacctgacgtgtcgacgcggccgcatgtcgttacataacttacggtaaatggccc
gcctggctgaccgcccaacgacccccgcccattgacgtcaataatgacgtatgttcccatagtaacgccaatagggactttccattgacgtca
atgggtggagtatttacggtaaactgcccacttggcagtacatcaagtgtatcatatgccaagtacgccccctattgacgtcaatgacggtaa
atggcccgcctggcattatgcccagtacatgaccttatgggactttcctacttggcagtacatctacgtattagtcatcgctattaccatggtgat
gcggttttggcagtacatcaatgggcgtggatagcggtttgactcacggggatttccaagtctccaccccattgacgtcaatgggagtttgtt
ttggcaccaaaatcaacgggactttccaaaatgtcgtaacaactccgccccattgacgcaaatgggcggtaggcgtgtacggtgggaggt
ctatataagcagagctcgtttagtgaaccgt

pACNR-CHIKV replicon sequence annotated

Feature	Location (nts)	Orientation with respect the CMV promoter	Type
5′ UTR	1 to 76	Right	5′ Untranslated region
nsp1	77 to 1,681	Right	CDS – nonstructural protein 1 – 59.5 kDa
nsp2	1,682 to 4,075	Right	CDS – nonstructural protein 2 – 89.6 kDa
nsp3	4,076 to 5,665	Right	CDS – nonstructural protein 3 – 57.5 kDa
nsp4	5,666 to 7,501	Right	CDS – nonstructural protein 3 – 68.2 kDa
SG promoter	7,472 to 7,504	Right	Promoter for the subgenomic RNA
Capsid	7,567 to 7,572	Right	CDS – capsid protein – 2 amino acids – 278 Da
E3 (partial)	7,573 to 7,578	Right	CDS –E3 - 2 amino acids – 202 Da
mKate2	7,579 to 8,286	Right	CDS – reporter gene mKate2 – 26.4 kDa
3′ UTR	8,287 to 9,024	Right	3′ Untranslated region that includes the viral polyA
HDV	9,025 to 9,108	Right	Hepatitis delta virus ribozyme
SV40 polyA	9,234 to 9,349	Right	Simian vacuolating virus 40 polyadenylation signal and transcription terminator
β-globin polyA	9,350 to 9,405	Right	β-globin polyadenylation signal and transcription terminator
p15A ori	10,132 to 10,676	Left	Origin of replication for *E. coli*
AmpR	10,841 to 11,701	Left	Ampicillin resistant gene for selection in *E. coli* – 31.5 kDa
AmpR promoter	11,702 to 11,806	Left	*E. coli* promoter for AmpR

CMV enhancer	11,855 to 12,158	Right	Enhancer for the Cytomegalovirus promoter for mammalian cell lines.
CMV promoter	12,159 to 12,362	Right	Cytomegalovirus transcription promoter for mammalian cell lines.

pACNR-Rep plasmid map

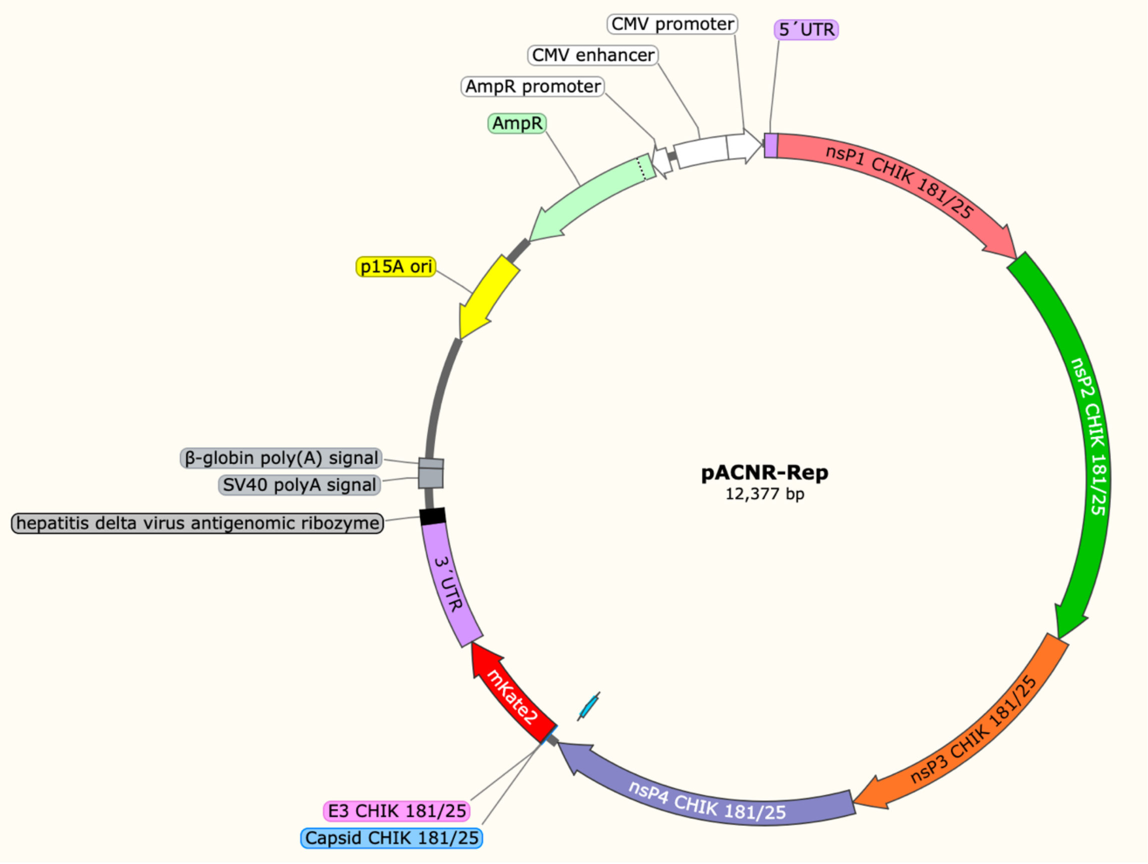

pVax-Helper plasmid sequence

GTACGCACTACAGCTACCTATTTTGCAGAAGCCGACAGCAGGTACCTAAATACCAATCAGCCATAATGGAG
TTTATCCCAACCCAAACTTTCTACAATAGGAGGTACCAGCCTCGACCTTGGACTCCGCGCCCTACTATCCAA
GTTATCAGACCCAGACCGCGTCCGCAAAGGAAAGCCGGGCAACTTGCCCAGCTGATCTCAGCAGTTAATA
AACTGACAATGCGCGCGGTACCTCAACAGAAGCCGCGCAAGAATCGGAAGAATAAGAAGCAAAAGCAAA
AGCAGCAGGCGCCACGAAACAACATGAATCAAAAGAAGCAGCCCCCTAAAAAGAAACCGGCTCAAAAGA
AAAAGAAGCCGGGCCGTAGAGAGAGAATGTGCATGAAAATCGAAAATGATTGCATCTTCGAAGTCAAGC
ATGAAGGTAAGGTAACAGGTTACGCGTGCTTGGTAGGGGACAAAGTAATGAAGCCAGCACACGTAAAGG
GGACCATCGATAATGCGGACCTGGCCAAATTGGCCTTCAAGCGGTCATCTAAGTACGACCTTGAATGCGC
GCAGATACCCGTGCACATGAAGTCCGACGCTTCGAAGTTCACCCATGAGAAACCGGAGGGGTACTACAAC
TGGCACCACGGAGCAGTACAGTACTCAGGAGGCCGGTTCACCATCCCTACAGGTGCGGGCAAACCAGGG
GACAGCGGTAGACCGATCTTCGACAACAAGGGGCGCGTGGTGGCCATAGTTTTAGGAGGAGCTAATGAA
GGAGCCCGTACAGCCCTCTCGGTGGTGACCTGGAACAAAGACATCGTCACGAAAATCACCCCTGAGGGG
GCCGAAGAGTGGAGTCTTGCCATTatggtgagcgagctgattaaggagaacatgcacatgaagctgtacatggagggcaccg
tgaacaaccaccacttcaagtgcacatccgagggcgaaggcaagccctacgagggcacccagaccatgagaatcaaggcggtcgaggg
cggccctctccccttcgccttcgacatcctggctaccagcttcatgtacggcagcaaaaccttcatcaaccacacccagggcatccccgacttc
tttaagcagtccttccccgagggcttcacatgggagagagtcaccacatacgaagacgggggcgtgctgaccgctacccaggacaccagc
ctccaggacggctgcctcatctacaacgtcaagatcagaggggtgaacttcccatccaacggccctgtgatgcagaagaaaacactcggct
gggaggcctccaccgagaccctgtaccccgctgacggcggcctggaaggcagagccgacatggccctgaagctcgtgggcgggggcc

acctgatctgcaacttgaagaccacatacagatccaagaaacccgctaagaacctcaagatgcccggcgtctactatgtggacagaagact
ggaaagaatcaaggaggccgacaaagagacctacgtcgagcagcacgaggtggctgtggccagatactgcgacctccctagcaaactg
gggcacagaaactttgacctacttaagttggcgggagacgttgagtccaaccctgggcccAGTCTTGCCATTCCAGTTATGTGC
CTGCTGGCAAATACCACGTTCCCCTGCTCCCAGCCCCCTTGCACACCCTGCTGCTACGAAAAAGAGCCGGA
GAAAACCCTGCGCATGCTAGAAGACAACGTCATGAGCCCCGGGTACTATCAGCTGCTACAAGCATCCTTA
ACATGTTCTCCCCGCCGCCAGCGACGCAGTATTAAGGACAACTTCAATGTCTATAAAGCCATAAGACCGTA
CCTAGCTCACTGTCCCGACTGTGGAGAAGGGCACTCGTGCCATAGTCCCGTAGCGCTAGAACGCATCAGA
AACGAAGCGACAGACGGGACGCTGAAAATCCAGGTTTCCTTGCAAATCGGAATAAAGACGGATGATAGC
CATGATTGGACCAAGCTGCGTTACATGGACAATCATATGCCAGCAGACGCAGAGAGGGCCAGGCTATTTG
TAAGAACGTCAGCACCGTGCACGATTACTGGAACAATGGGACACTTCATCCTGGCCCGATGTCCGAAAGG
AGAAACTCTGACGGTGGGATTCACTGACGGTAGGAAGATCAGTCACTCATGTACGCACCCATTTCACCACG
ACCCTCCTGTGATAGGCCGGGAAAAATTTCATTCCCGACCGCAGCACGGTAGAGAACTACCTTGCAGCAC
GTACGCGCAGAGCACCGCTGCAACTGCCGAGGAGATAGAGGTACATATGCCCCCAGACACCCCAGATCG
CACATTGATGTCACAACAGTCCGGTAATGTAAAGATCACAGTCAATAGTCAGACGGTGCGGTACAAGTGT
AATTGCGGTGACTCAAATGAAGGACTAACCACTACAGACAAAGTGATTAATAACTGCAAGGTTGATCAATG
CCATGCCGCGGTCACCAATCACAAAAAATGGCAGTATAATTCCCCTCTGGTCCCGCGTAATGCTGAACTCG
GGGACCGAAAAGGAAAAGTTCACATTCCGTTTCCTCTGGCAAATGTGACATGCAGGGTGCCTAAGGCAAG
GAACCCCACCGTGACGTACGGAAAAAACCAAGTCATCATGCTGCTGTATCCTGACCACCCAACGCTCCTGT
CCTACCGGAATATGGGAGAAGAACCAAACTATCAAGAAGAGTGGGTGACGCATAAGAAGGAGATCAGGT
TAACCGTGCCGACTGAAGGGCTCGAGGTCACGTGGGGCAACAACGAGCCGTACAAGTATTGGCCGCAGTT
ATCCACAAACGGTACAGCCCACGGCCACCCGCATGAGATAATTTTGTATTATTATGAGCTGTACCCTACTAT
GACTGTGGTAGTTGTGTCAGTGGCCTCGTTCGTACTCCTGTCGATGGTGGGTGTGGCAGTGGGGATGTGCA
TGTGTGCACGACGCAGATGCATTACACCGTACGAACTGACACCAGGAGCTACCGTCCCTTTCCTGCTTAGC
CTAATATGCTGCATTAGAACAGCTAAAGCGGCCACATACCAAGAGGCTGCGGTATACCTGTGGAACGAGC
AGCAGCCTTTGTTTTGGCTGCAAGCCCTTATTCCGCTGGCAGCCCTGATTGTCCTATGCAACTGTCTGAGAC
TCTTACCATGCTTTTGTAAAACGTTGACTTTTTAGCCGTAATGAGCGTCGGTGCCCACACTGTGAGCGCGT
ACGAACACGTAACAGTGATCCCGAACACGGTGGGAGTACCGTATAAGACTCTAGTCAACAGACCGGGCTA
CAGCCCCATGGTACTGGAGATGGAGCTTCTGTCAGTCACTTTGGAGCCAACGCTATCGCTTGATTACATCA
CGTGCGAGTATAAAACCGTCATCCCGTCTCCGTACGTGAAATGCTGCGGTACAGCAGAGTGCAAGGACAA
GAGCCTACCTGATTACAGCTGTAAGGTCTTCACCGGCGTCTACCCATTCATGTGGGGCGGCGCCTACTGCT
TCTGCGACACTGAAAATACGCAATTGAGCGAAGCACATGTGGAGAAGTCCGAATCATGCAAAACAGAATT
TGCATCAGCATATAGGGCTCATACCGCATCCGCATCAGCTAAGCTCCGCGTCCTTTACCAAGGAAATAATG
TTACTGTATCTGCTTATGCAAACGGCGATCATGCCGTCACAGTTAAGGACGCTAAATTCATTGTGGGGCCA
ATGTCTTCAGCCTGGACACCTTTTGACAATAAAATCGTGGTGTACAAAGGCGACGTCTACAACATGGACTA
CCCGCCCTTCGGCGCAGGAAGACCAGGACAATTTGGCGACATCCAAAGTCGCACGCCTGAGAGCGAAGA
CGTCTATGCTAACACACAACTGGTACTGCAGAGACCGTCCGCGGGTACGGTGCACGTGCCGTACTCTCAG
GCACCATCTGGCTTCAAGTATTGGCTAAAAGAACGAGGGGCGTCGCTGCAGCACACAGCACCATTTGGCT
GTCAAATAGCAACAAACCCGGTAAGAGCGATGAACTGCGCCGTAGGGAACATGCCTATCTCCATCGACAT
ACCGGACGCGGCCTTCACTAGGGTCGTCGACGCGCCATCTTTAACGGACATGTCGTGTGAGGTACCAGCC
TGCACCCACTCCTCAGACTTTGGGGGCGTAGCCATCATTAAATATGCAGCCAGCAAGAAAGGCAAGTGTG
CGGTGCATTCGATGACTAACGCCGTCACTATTCGGGAAGCTGAAATAGAAGTAGAAGGGAACTCTCAGTT
GCAAATCTCTTTTTCGACGGCCCTAGCCAGCGCCGAATTCCGCGTACAAGTCTGTTCTACACAAGTACACT
GTGCAGCCGAGTGCCATCCACCGAAAGACCATATAGTCAATTACCCGGCGTCACACACCACCCTCGGGGT
CCAAGACATTTCCGTTACGGCGATGTCATGGGTGCAGAAGATCACGGGAGGTGTGGGACTGGTTGTCGCT
GTTGCAGCACTGATCCTAATCGTGGTGCTATGCGTGTCGTTTAGCAGGCACTAACTTGACAACTAGGTACG

AAGGTATATGTGTCCCCTAAGAGACACACCACATATAGCTAAGAATCAATAGATAAGTATAGATCAAAGG
GCTGAACAACCCCTGAATAGTAACAAAATATAAAAATCAACAAAAATCATAAAATAGAAAACCAGAAACA
GAAGTAGGTAAGAAGGTATATGTGTCCCCTAAGAGACACACCATATATAGCTAAGAATCAATAGATAAGT
ATAGATCAAAGGGCTGAATAACCCCTGAATAATAACAAAATATAAAAATCAATAAAAATCATAAAATAGAA
AACCATAAACAGAAGTAGTTCAAAGGGCTATAAAACCCCTGAAAAGTAACAAAACATAAAACTAATAAAA
ATCAAATGAATACCATAATTGGCAATCGGAAGAGATGTAGGTACTTAAGCTTCCTAAAAGCAGCCGAACTC
GCTTTGAGATGTAGGCGTAGCACACCGAACTCTTCCATAATTCTCCGAACCCACAGGGACGTAGGAGATGT
TCAAAGTGGCTATAAAACCCTGAACAGTAATAAAACATAAAATTAATAAGGATCAAATGAGTACCATAATT
GGCAAACGGAAGAGATGTAGGTACTTAAGCTTCCTAAAAGCAGCCGAACTCACTTTGAGATGTAGGCATA
GCATACCGAACTCTTCCACAATTCTCCGTACCCATAGGGACGTAGGAGATGTTATTTTGTTTTTAATATTTCA
AACTGTGCCTTCTAGTTGCCAGCCATCTGTTGTTTGCCCCTCCCCCGTGCCTTCCTTGACCCTGGAAGGTGC
CACTCCCACTGTCCTTTCCTAATAAAATGAGGAAATTGCATCGCATTGTCTGAGTAGGTGTCATTCTATTCTG
GGGGGTGGGGTGGGGCAGGACAGCAAGGGGGAGGATTGGGAAGACAATAGCAGGCATGCTGGGGATGC
GGTGGGCTCTATGGCTTCTACTGGGCGGTTTTATGGACAGCAAGCGAACCGGAATTGCCAGCTGGGGCGC
CCTCTGGTAAGGTTGGGAAGCCCTGCAAAGTAAACTGGATGGCTTTCTCGCCGCCAAGGATCTGATGGCG
CAGGGGATCAAGCTCTGATCAAGAGACAGGATGAGGATCGTTTCGCATGATTGAACAAGATGGATTGCAC
GCAGGTTCTCCGGCCGCTTGGGTGGAGAGGCTATTCGGCTATGACTGGGCACAACAGACAATCGGCTGCT
CTGATGCCGCCGTGTTCCGGCTGTCAGCGCAGGGGCGCCCGGTTCTTTTTGTCAAGACCGACCTGTCCGGT
GCCCTGAATGAACTGCAAGACGAGGCAGCGCGGCTATCGTGGCTGGCCACGACGGGCGTTCCTTGCGCA
GCTGTGCTCGACGTTGTCACTGAAGCGGGAAGGGACTGGCTGCTATTGGGCGAAGTGCCGGGGCAGGAT
CTCCTGTCATCTCACCTTGCTCCTGCCGAGAAAGTATCCATCATGGCTGATGCAATGCGGCGGCTGCATAC
GCTTGATCCGGCTACCTGCCCATTCGACCACCAAGCGAAACATCGCATCGAGCGAGCACGTACTCGGATG
GAAGCCGGTCTTGTCGATCAGGATGATCTGGACGAAGAGCATCAGGGGCTCGCGCCAGCCGAACTGTTCG
CCAGGCTCAAGGCGAGCATGCCCGACGGCGAGGATCTCGTCGTGACCCATGGCGATGCCTGCTTGCCGAA
TATCATGGTGGAAAATGGCCGCTTTTCTGGATTCATCGACTGTGGCCGGCTGGGTGTGGCGGACCGCTATC
AGGACATAGCGTTGGCTACCCGTGATATTGCTGAAGAGCTTGGCGGCGAATGGGCTGACCGCTTCCTCGT
GCTTTACGGTATCGCCGCTCCCGATTCGCAGCGCATCGCCTTCTATCGCCTTCTTGACGAGTTCTTCTGAATT
ATTAACGCTTACAATTTCCTGATGCGGTATTTTCTCCTTACGCATCTGTGCGGTATTTCACACCGCATACAGG
TGGCACTTTTCGGGGAAATGTGCGCGGAACCCCTATTTGTTTATTTTTCTAAATACATTCAAATATGTATCCG
CTCATGAGACAATAACCCTGATAAATGCTTCAATAATAGCACGTGCTAAAACTTCATTTTTAATTTAAAAGG
ATCTAGGTGAAGATCCTTTTTGATAATCTCATGACCAAAATCCCTTAACGTGAGTTTTCGTTCCACTGAGCGT
CAGACCCCGTAGAAAAGATCAAAGGATCTTCTTGAGATCCTTTTTTTCTGCGCGTAATCTGCTGCTTGCAAA
CAAAAAAACCACCGCTACCAGCGGTGGTTTGTTTGCCGGATCAAGAGCTACCAACTCTTTTTCCGAAGGTA
ACTGGCTTCAGCAGAGCGCAGATACCAAATACTGTCCTTCTAGTGTAGCCGTAGTTAGGCCACCACTTCAA
GAACTCTGTAGCACCGCCTACATACCTCGCTCTGCTAATCCTGTTACCAGTGGCTGCTGCCAGTGGCGATA
AGTCGTGTCTTACCGGGTTGGACTCAAGACGATAGTTACCGGATAAGGCGCAGCGGTCGGGCTGAACGGG
GGGTTCGTGCACACAGCCCAGCTTGGAGCGAACGACCTACACCGAACTGAGATACCTACAGCGTGAGCTA
TGAGAAAGCGCCACGCTTCCCGAAGGGAGAAAGGCGGACAGGTATCCGGTAAGCGGCAGGGTCGGAAC
AGGAGAGCGCACGAGGGAGCTTCCAGGGGGAAACGCCTGGTATCTTTATAGTCCTGTCGGGTTTCGCCAC
CTCTGACTTGAGCGTCGATTTTTGTGATGCTCGTCAGGGGGGCGGAGCCTATGGAAAAACGCCAGCAACG
CGGCCTTTTTACGGTTCCTGGGCTTTTGCTGGCCTTTTGCTCACATGTTCTTGACTCTTCGCGATGTACGGGC
CAGATATACGCGTTGACATTGATTATTGACTAGTTATTAATAGTAATCAATTACGGGGTCATTAGTTCATAG
CCCATATATGGAGTTCCGCGTTACATAACTTACGGTAAATGGCCCGCCTGGCTGACCGCCCAACGACCCCC
GCCCATTGACGTCAATAATGACGTATGTTCCCATAGTAACGCCAATAGGGACTTTCCATTGACGTCAATGG
GTGGACTATTTACGGTAAACTGCCCACTTGGCAGTACATCAAGTGTATCATATGCCAAGTACGCCCCCTATT

GACGTCAATGACGGTAAATGGCCCGCCTGGCATTATGCCCAGTACATGACCTTATGGGACTTTCCTACTTG
GCAGTACATCTACGTATTAGTCATCGCTATTACCATGGTGATGCGGTTTTGGCAGTACATCAATGGGCGTG
GATAGCGGTTTGACTCACGGGGATTTCCAAGTCTCCACCCCATTGACGTCAATGGGAGTTTGTTTTGGCAC
CAAAATCAACGGGACTTTCCAAAATGTCGTAACAACTCCGCCCCATTGACGCAAATGGGCGGTAGGCGTG
TACGGTGGGAGGTCTATATAAGCAGAGCT

pACNR-CHIKV replicon sequence annotated

Feature	Location (nts)	Orientation with respect the CMV promoter	Type
5′ UTR	1 to 65	Right	5′ Untranslated region
Capsid	66 to 848	Right	CDS – capsid protein – 29.6 kDa
E3 (partial)	849 to 860	Right	CDS – partial sequence of the E3 chaperone – This allows the correct autoproteolytic cleavage between C and mKate2 – 402 Da
mKate2	861 to 1,556	Right	CDS – reporter gene mKate2 – 26.1 kDa
FMV2	1,557 to 1,607	Right	CDS – Foot and mouse disease virus cleave site to separate mKate 2 from E3.
E3	1,608 to 1,799	Right	CDS – E3 protein – 7.3 kDa
E2	1,800 to 3,068	Right	CDS – E2 glycoprotein – 47.5 kDa
6K	3,069 to 3,251	Right	CDS – 6K protein – 6.8 kDa
E1	3,252 to 4,571	Right	CDS – E1glycoprotein – 47.5 kDa
3′ UTR	4,572 to 5,297	Right	3′ Untranslated region that includes the viral polyA
β-globin polyA	5,298 to 5,522	Right	β-globin polyadenylation signal and transcription terminator
NeoR/KanR	5,695 to 6,489	Right	Aminoglycoside phosphotransferase confers resistance to neomycin, kanamycin, and G418 (Geneticin®) for selection in *E. coli* – 29.0 kDa
pUC ori	6,815 to 7,403	Right	Origin of replication for *E. coli*
CMV enhancer	7,504 to 7,883	Right	Enhancer for the Cytomegalovirus promoter for mammalian cell lines.
CMV promoter	7,884 to 8,087	Right	Cytomegalovirus transcription promoter for mammalian cell lines.

pACNR-Rep plasmid map

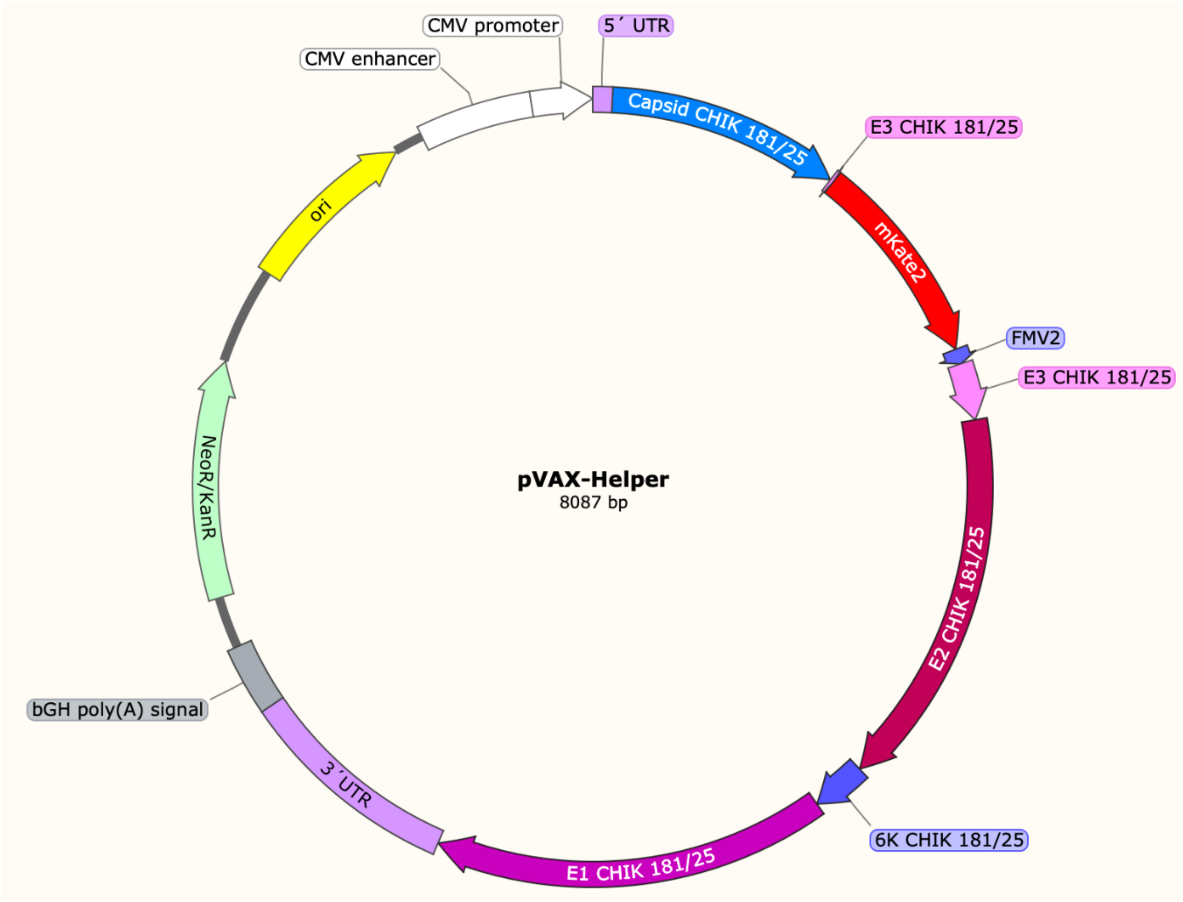

Supplement: Supplementary file 1 [file viruses-15-00132-s001.zip › viruses-2057418-supplementary.pdf]
